# Supplementary material for: Lithium and Vanadium Intercalation into Bilayer V2Se2O: Ferrimagnetic–Ferroelastic Multiferroics and Anomalous and Spin Transport
Source: Adv Sci (Weinh). 2025 Nov 7;13(5):e12533. doi: 10.1002/advs.202512533 (PMC12850265; doi:10.1002/advs.202512533)
Supplement: Supplementary file 1 — Supporting Information [file ADVS-13-e12533-s001.pdf]

Supporting Information

Lithium and Vanadium Intercalation into Bilayer V_2Se_2O: Ferrimagnetic-Ferroelastic Multiferroics and Anomalous and Spin Transport

Long Zhang, Yuxin Liu, Junfeng Ren, Guangqian Ding, Xiaotian Wang, Guangxin Ni,* Guoying Gao,* and Zhenxiang Cheng**

L. Zhang, G. Gao

School of Physics and Wuhan National High Magnetic Field Center
Huazhong University of Science and Technology, Wuhan 430074, China
E-mail: guoying_gao@mail.hust.edu.cn

Y. Liu, J. Ren

School of Physics and Electronics, Shandong Provincial Engineering and Technical Center of Light Manipulations & Institute of Materials and Clean Energy
Shandong Normal University, Jinan 250358, China

G. Ding

School of Sciences and Institute for Advanced Sciences
Chongqing University of Posts and Telecommunications, Chongqing 400065, China

X. Wang, Z. Cheng

Institute for Superconducting and Electronic Materials, Faculty of Engineering and Information Sciences
University of Wollongong, Wollongong 2500, Australia
E-mail: xiaotianw@uow.edu.au; cheng@uow.edu.au

G. Ni

Department of Physics
Florida State University, Tallahassee, FL 32306, USA
E-mail: guangxin.ni@magnet.fsu.edu

G. Ni

National High Magnetic Field Laboratory, Tallahassee, FL 32310, USA

1. Magnetic Anisotropy

To further clarify atomic and orbital origins, we analyze the V-*d* and Se-*p* intra-orbital hybridization using the second-order perturbation theory,[1] the system's Hamiltonian (H) can be divided into an unperturbed component (H_0) and a perturbation term (λV) as follows,

$$H = H_0 + \lambda V \tag{1}$$

thus, the energy and wave function can be expanded and brought into the Schrödinger equation as,

$$H_0 |n^{(2)}\rangle + V|n^{(1)}\rangle = E_n^{(0)} |n^{(2)}\rangle + E_n^{(1)} |n^{(1)}\rangle + E_n^{(2)} |n^{(0)}\rangle \tag{2}$$

then the first-order wave function can be written as,

$$|n^{(1)}\rangle = \sum_{m \neq n} \frac{\langle m^{(0)}|V|n^{(0)}\rangle}{E_n^{(0)} - E_m^{(0)}} |m^{(0)}\rangle \tag{3}$$

and the second-order energy is attained as,

$$E_n^{(2)} = \sum_{m \neq n} \frac{|\langle m^{(0)}|V|n^{(0)}\rangle|^2}{E_n^{(0)} - E_m^{(0)}} \tag{4}$$

The magnetic anisotropy energy arises from spin-orbit coupling (SOC) and can be expressed as,

$$\mathrm{MAE} = \xi^2 \sum_{o,u} \sum_{\alpha,\beta} (1 - 2\delta_{\alpha\beta}) \frac{|\langle o^{\alpha}|L_x|u^{\beta}\rangle|^2 - |\langle o^{\alpha}|L_z|u^{\beta}\rangle|^2}{E_u^{\beta} - E_o^{\alpha}} \tag{5}$$

in which ξ represents the SOC constant, $\delta_{\alpha\beta}$ is the Kronecker delta, which is 0 elsewhere and 1 when $\alpha = \beta$. α and β stand for the spin orientations, and o and u are the occupied and unoccupied states, respectively.

2. Magnetic Critical Temperature

Specifically, the specific heat capacity[2] is defined as,

$$C_V = \frac{<E^2> - <E>^2}{k_B T^2} \tag{6}$$

where $(<E^2> - <E>^2)$ denotes the energy variance or fluctuations within the thermodynamic system, k_B is the Boltzmann constant, and the square of temperature

(T) normalizes the energy fluctuations. Thus, the specific heat capacity C_V is proportional to the energy fluctuations and manifests the response sensitivity of the system to the temperature changes.

The energies of the considered magnetic configurations are listed in Table S1. The exchange coupling parameters were derived as follows: (1) J_{int} can be calculated as J_{int} = [E(AFM1-AFM) – E(AFM1-FM)]/16. (2) J_1 can be obtained by J_1 = [E(AFM1-FM) – E(FM-FM)]/64. (3) J_{2x} was determined using the following relationships: E(AFM1-FM) – E(AFM4-FM) = $32J_1 - 32J_{2x} - 32J_{2y}$, and E(AFM5-FM) – E(AFM6-FM) = $16J_{2y} - 16J_{2x}$. Combining these, E(AFM1-FM) – E(AFM4-FM) + 2*[E(AFM5-FM) – E(AFM6-FM)] = $36J_1 - 64J_{2x}$. With J_1 value known, J_{2x} was then solved for. (4) Subsequently, J_{2x} was calculated using the known value of J_{2y} and the equation E(AFM5-FM) – E(AFM6-FM) = $16J_{2y} - 16J_{2x}$. (5) Finally, J_{11} was extracted from the expression: E(AFM5-FM) + E(AFM6-FM) – 2*E(AFM4-FM) = $-16J_{2x} - 16J_{2y} + 64J_{11}$.

The calculated critical temperature for the Li-intercalated bilayer V_2Se_2O (358 K) is consistent with experimental values reported for KV_2Se_2O and $Rb_{1-\delta}V_2Te_2O$ (≥ 300 K).[3,4] Similarly, the above-RT values of V-intercalated and pristine bilayers (773 and 960 K, respectively) agree with those of related compounds, such as V_2Te_2O (740 K)[5] and V_2SeTeO (510 K).[6] Furthermore, the trend observed upon intercalation in the tellurium-based system (pristine V_2Te_2O to $Rb_{1-\delta}V_2Te_2O$) appears to align well with that found in the selenium-based system (from pristine V_2Se_2O to Li- and K-intercalated sample). The reliability of our Monte Carlo (MC) simulation approach is supported by previous validation studies. Our calculated Curie temperature (T_C) for CrI_3 monolayer (48 K)[7] closely matched the experimental value (45 K).[8] Similarly, our simulated T_Cs for MnSeTe and MnSTe (178 and 141 K)[7] are consistent with those reported by Yang's and Fert's groups (170 and 140 K, respectively).[9]

3. Equilibrium Transport Performance

For a quantitative comparison of transport performance, we calculate several essential metrics. The spin-dependent transmission coefficient is given by,

$$T_{\uparrow(\downarrow)}(E) = Tr\left[\Gamma_L(E)G^R(E)\Gamma_R(E)G^A(E)\right]_{\uparrow(\downarrow)} \tag{7}$$

$G^{R/A}$ is the retarded/advanced Green's function of the central region. $\Gamma_{L/R}(E)$ is the coupling matrix of the left/right electrode, which can be described as,

$$\Gamma_{L/R}(E) = i\left[\sum_{L/R}^{R}(E) - \sum_{L/R}^{A}(E)\right] \tag{8}$$

The conductance $G_{\uparrow(\downarrow)}$ can be defined as,

$$G_{\uparrow(\downarrow)} = \frac{e^2}{h}T_{\uparrow(\downarrow)} \tag{9}$$

The transmission coefficients T_{P} and T_{AP} can be expressed as,

$$T_{\mathrm{P(AP)}} = \sum_{\vec{k}_{//}} T_{\mathrm{P(AP)}}(\vec{k}_{//}) / N_k \tag{10}$$

where the in-plane wave vector $\vec{k}_{//} = (k_x, k_y)$ is perpendicular to the transport direction, and N_k is the number of k points.

4. Temperature Effect on Spin Current

To evaluate temperature-dependent spin transport performance, we consider the thermal spin current is written as,

$$I_{\uparrow(\downarrow)} = \frac{e}{h}\int_{-\infty}^{\infty}\left\{T_{\uparrow(\downarrow)}(E)\left[f_L(E,T_L) - f_R(E,T_R)\right]\right\}\mathrm{d}E \tag{11}$$

where T_{L} and T_{R} are the temperatures of the left and right electrodes, the temperature difference is set as $\Delta T = T_L - T_R$. The $f_L(E,T_L)$ and $f_R(E,T_R)$ are the Fermi-Dirac distribution for the left and right electrodes, respectively. e is the electron charge, h represents Planck's constant, and ↑, ↓ are spin indexes. The energy- and temperature-dependent Fermi-Dirac distribution is obtained as,

$$f_{L(R)}(E,T_L) = \frac{1}{e^{E/kT_{L(R)}} + 1} \tag{12}$$

and the Fermi-Dirac distribution difference is calculated as,

$$\Delta f(E,T) = f_L(E,T_L) - f_R(E,T_R) \tag{13}$$

The thermal current is the integral over energy of the product of the transmission coefficient and the Δf. Only regions where both transmission coefficient and the Δf are non-zero contribute meaningfully to the current. Accordingly, the thermal spin filtering efficiency η under a temperature gradient can be defined as,

$$\eta = \frac{|I_\uparrow| - |I_\downarrow|}{|I_\uparrow| + |I_\downarrow|} \times 100\% \tag{14}$$

where $I_\uparrow$ and $I_\downarrow$ are the total currents in spin-up and spin-down channels, respectively, and positive and negative spin filtering efficiencies represent the dominance of spin-up and spin-down currents, respectively. The thermal magnetoresistance (MR) ratio is calculated by,

$$\mathrm{MR_{thermal}} = \frac{I_{\mathrm{P}} - I_{\mathrm{AP}}}{I_{\mathrm{AP}}} \times 100\% \tag{15}$$

in which the I_{P} and I_{AP} are the total thermal currents in the parallel (P) and antiparallel (AP) magnetization alignments, respectively. These metrics allow for the evaluation of how effectively a device can filter spin currents and respond to temperature-induced changes in magnetization alignments.

5. Experimental Section

Van der Waals interactions were incorporated using the DFT-D3 method.[10] The climbing image nudged elastic band (CI-NEB) method was implemented for calculating the energy barriers.[11] The VASPBERRY code was utilized for calculating Berry curvature from the wave function,[12] and the Wannier90[13] and WannierTools[14] packages were used to calculate the anomalous Hall conductivity (AHC). The Metropolis MC methods for magnetic critical temperatures were simulated within the MCSOLVER package.[15] A 100 × 100 × 1 supercell and 10^5 steps were utilized during MC simulations. For atomic/orbital-resolved magnetic anisotropy energy (MAE) calculations incorporating SOC, the parameters LORBMOM = .T. and LORBIT = 11 were set to enable the computation of orbital moments and output the contributions from individual atoms and orbits. The P4VASP[16] and VASPKIT[17] packages were used for pre- and post-processing the data from VASP. The full lattice

and atomic relaxations were performed. The cutoff energy was set as 600 eV, and the convergences tolerances for force and energy were 0.001 eV Å^{-1} and 10^{-7} eV, respectively. The Γ-centered 14 × 14 × 1 Monkhorst-Pack grid was utilized, and a 15 Å vacuum was included to eliminate the interaction from the periodic structures.

For spin transport calculations, the cutoff energy was considered 150 hartree, and the double-*z* plus polarization (DZP) basis set was employed. The 15 × 15 × 150 and 150 × 150 *k*-meshes were utilized for self-consistent and transmission calculations, respectively.

---

[1] D.-s. Wang, R. Wu, A. J. Freeman, First-principles theory of surface magnetocrystalline anisotropy and the diatomic-pair model, *Phys. Rev. B* **1993**, *47*, 14932.

[2] C. Huang, J. Feng, J. Zhou, H. Xiang, K. Deng, E. Kan, Ultra-High-Temperature Ferromagnetism in Intrinsic Tetrahedral Semiconductors, *J. Am. Chem. Soc.* **2019**, *141*, 12413.

[3] B. Jiang, M. Hu, J. Bai, Z. Song, C. Mu, G. Qu, W. Li, W. Zhu, H. Pi, Z. Wei, Y.-J. Sun, Y. Huang, X. Zheng, Y. Peng, L. He, S. Li, J. Luo, Z. Li, G. Chen, H. Li, H. Weng, T. Qian, A metallic room-temperature *d*-wave altermagnet, *Nat. Phys.* **2025**, *21*, 754.

[4] F. Zhang, X. Cheng, Z. Yin, C. Liu, L. Deng, Y. Qiao, Z. Shi, S. Zhang, J. Lin, Z. Liu, M. Ye, Y. Huang, X. Meng, C. Zhang, T. Okuda, K. Shimada, S. Cui, Y. Zhao, G.-H. Cao, S. Qiao, J. Liu, C. Chen, Crystal-symmetry-paired spin–valley locking in a layered room-temperature metallic altermagnet candidate, *Nat. Phys.* **2025**, *21*, 760.

[5] Q. Cui, Y. Zhu, X. Yao, P. Cui, H. Yang, Giant spin-Hall and tunneling magnetoresistance effects based on a two-dimensional nonrelativistic antiferromagnetic metal, *Phys. Rev. B* **2023**, *108*, 024410.

[6] A. ullah, D. Bezzerga, J. Hong, Giant spin seebeck effect with highly polarized spin current generation and piezoelectricity in flexible V_2SeTeO altermagnet at room temperature, *Mater. Today Phys.* **2024**, *47*, 101539.

[7] L. Zhang, Y. Zhao, Y. Liu, G. Gao, High spin polarization, large perpendicular magnetic anisotropy and room-temperature ferromagnetism by biaxial strain and carrier doping in Janus MnSeTe and MnSTe, *Nanoscale* **2023**, *15*, 18910.

[8] B. Huang, G. Clark, E. Navarro-Moratalla, D. R. Klein, R. Cheng, K. L. Seyler, D. Zhong, E. Schmidgall, M. A. McGuire, D. H. Cobden, W. Yao, D. Xiao, P. Jarillo-Herrero, X. Xu, Layer-dependent ferromagnetism in a van der Waals crystal down to the monolayer limit, *Nature* **2017**, *546*, 270.

[9] J. Liang, W. Wang, H. Du, A. Hallal, K. Garcia, M. Chshiev, A. Fert, H. Yang, Very large Dzyaloshinskii-Moriya interaction in two-dimensional Janus manganese dichalcogenides and its application to realize skyrmion states, *Phys. Rev. B* **2020**, *101*, 184401.

[10] S. Grimme, J. Antony, S. Ehrlich, H. Krieg, A consistent and accurateab initioparametrization of density functional dispersion correction (DFT-D) for the 94 elements H-Pu, *J. Chem. Phys.* **2010**, *132*, 154104.

[11] G. Henkelman, B. P. Uberuaga, H. Jónsson, A climbing image nudged elastic band method for finding saddle points and minimum energy paths, *J. Chem. Phys.* **2000**, *113*, 9901.
[12] S.-W. Kim, H.-J. Kim, S. Cheon, T.-H. Kim, Circular Dichroism of Emergent Chiral Stacking Orders in Quasi-One-Dimensional Charge Density Waves, *Phys. Rev. Lett.* **2022**, *128*, 046401.
[13] A. A. Mostofi, J. R. Yates, G. Pizzi, Y.-S. Lee, I. Souza, D. Vanderbilt, N. Marzari, An updated version of wannier90: A tool for obtaining maximally-localised Wannier functions, *Comput. Phys. Commun.* **2014**, *185*, 2309.
[14] Q. Wu, S. Zhang, H.-F. Song, M. Troyer, A. A. Soluyanov, WannierTools: An open-source software package for novel topological materials, *Comput. Phys. Commun.* **2018**, *224*, 405.
[15] L. Liu, X. Ren, J. Xie, B. Cheng, W. Liu, T. An, H. Qin, J. Hu, Magnetic switches via electric field in BN nanoribbons, *Appl. Surf. Sci.* **2019**, *480*, 300.
[16] https://github.com/orest-d/p4vasp.
[17] V. Wang, N. Xu, J.-C. Liu, G. Tang, W.-T. Geng, VASPKIT: A user-friendly interface facilitating high-throughput computing and analysis using VASP code, *Comput. Phys. Commun.* **2021**, *267*, 108033.

Supporting Figures and Tables

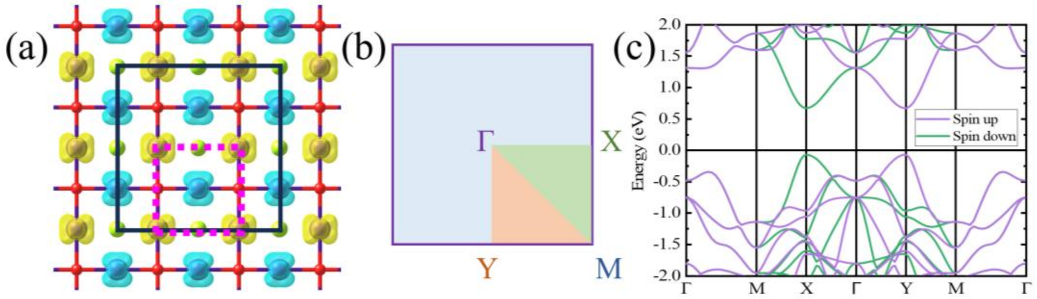

Figure S1. The charge density of the altermagnetic (AM) configuration (a), the high-symmetry points in the first Brillouin zone (b) and the spin-resolved band structures (c) for V_2Se_2O monolayer.

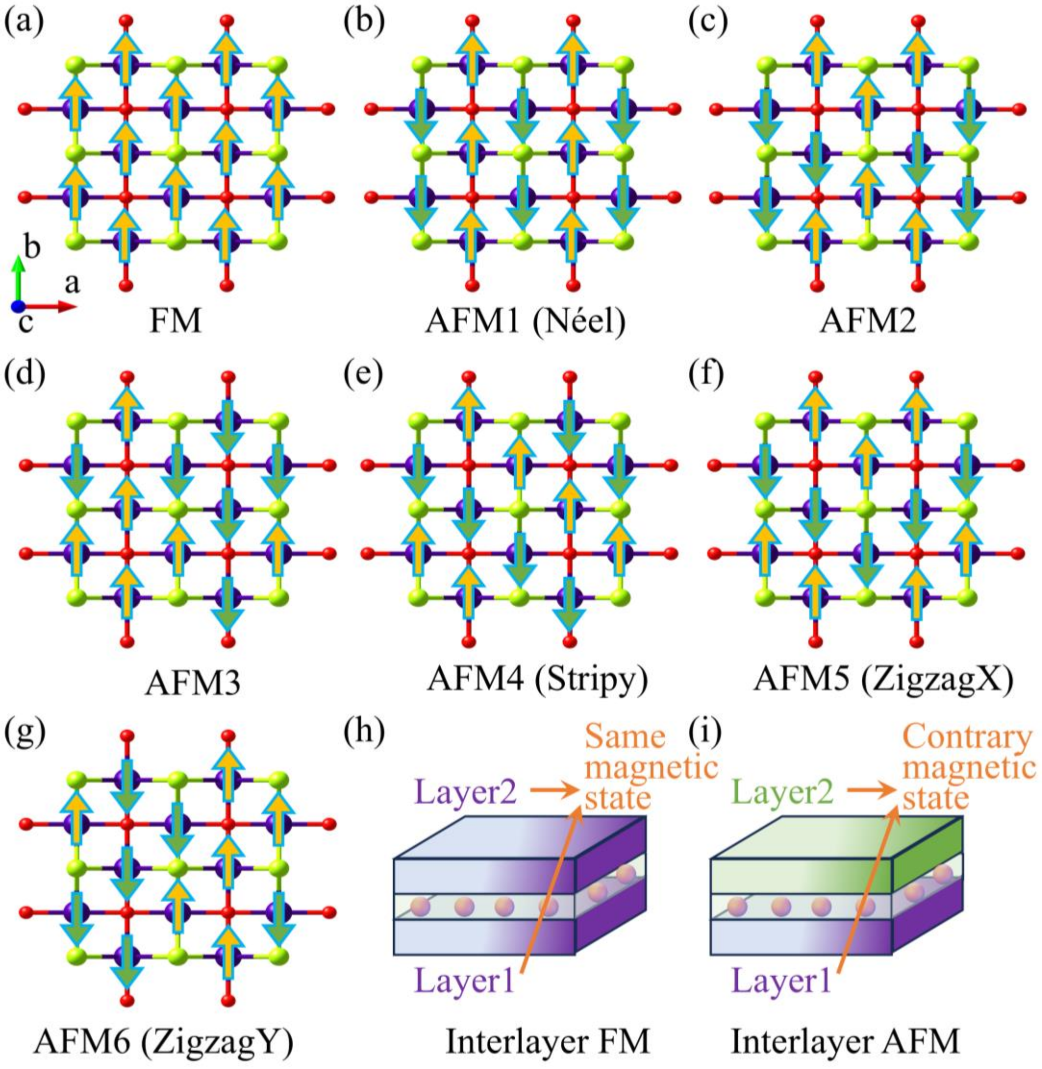

Figure S2. The intralayer ferromagnetic (FM) (a) and antiferromagnetic (AFM) (b-g), and interlayer FM (h) and AFM (i) configurations for V_2Se_2O bilayer with and without intercalation.

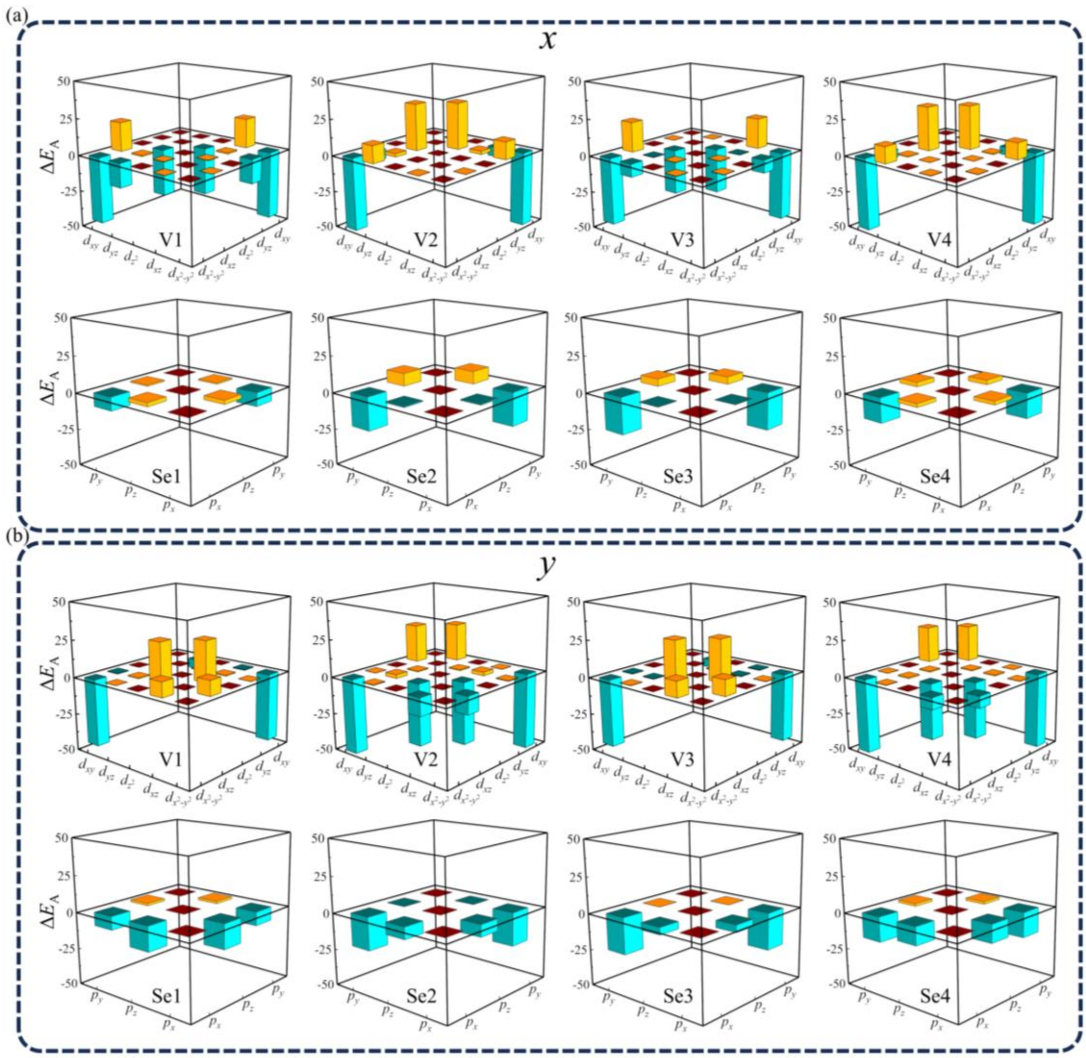

Figure S3. The V-*d*- and Se-*p*-orbital-resolved ΔE_As for V_2Se_2O bilayer with Li intercalation with magnetization axis along *x*- (a) and *y*- (b) directions. The ΔE_As are with reference to the states with magnetization axis along *z*-direction.

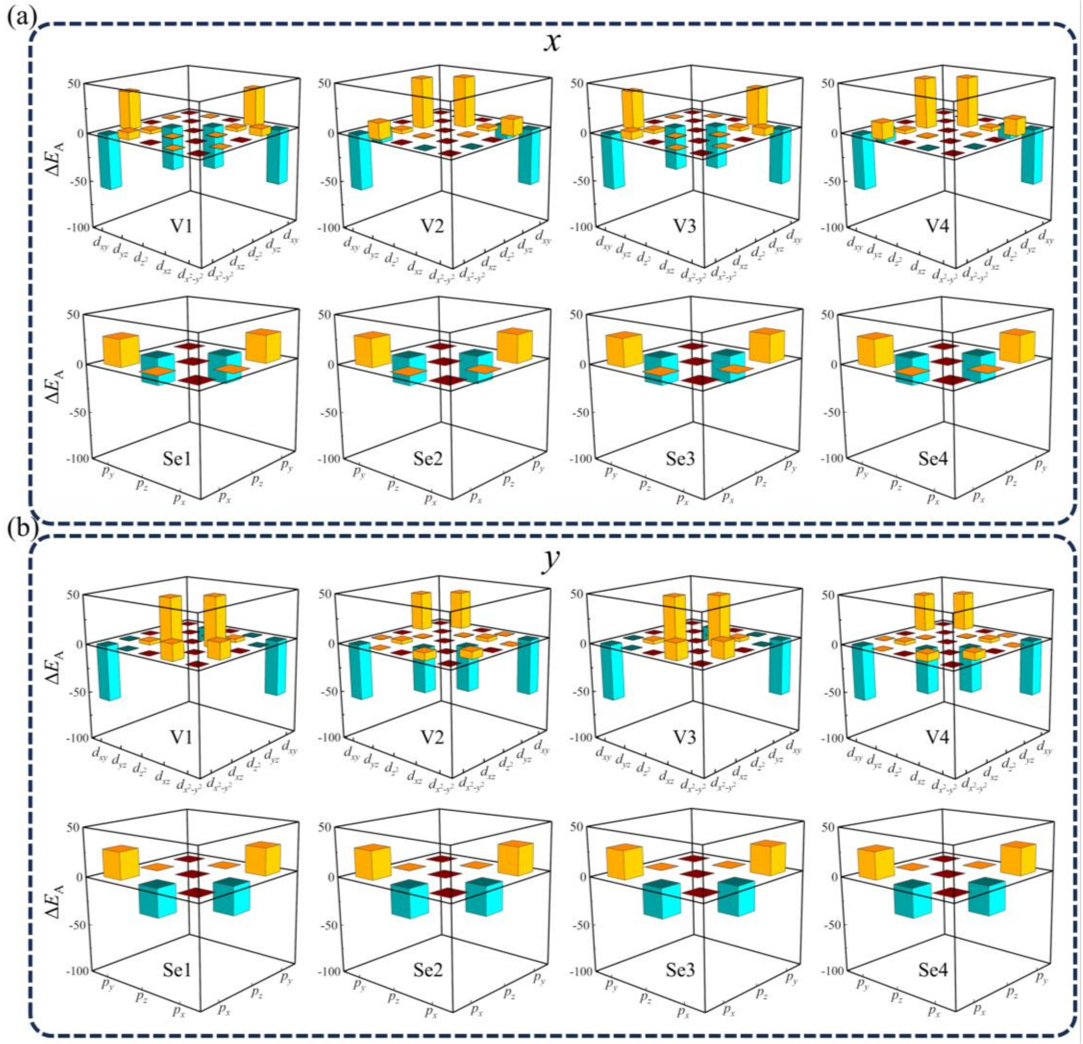

Figure S4. The V-*d*- and Se-*p*-orbital-resolved ΔE_As for V_2Se_2O bilayer with no intercalation with magnetization axis along *x*- (a) and *y*- (b) directions. The ΔE_As are with reference to the states with magnetization axis along *z*-direction.

Table S1. The energies of magnetic states based on Heisenberg spin Hamiltonian among the 2 × 2 × 1 supercell of V_2Se_2O bilayer with and without intercalations.

Magnetic states	Energy form
FM-FM	$E_0 - 32J_1 - 16J_{2x} - 16J_{2y} - 32J_{11} - 8J_{int} - 16A$
FM-AFM	$E_0 - 32J_1 - 16J_{2x} - 16J_{2y} - 32J_{11} + 8J_{int} - 16A$
AFM1-FM	$E_0 + 32J_1 - 16J_{2x} - 16J_{2y} - 32J_{11} - 8J_{int} - 16A$
AFM1-AFM	$E_0 + 32J_1 - 16J_{2x} - 16J_{2y} - 32J_{11} + 8J_{int} - 16A$
AFM2-FM	$E_0 + 0J_1 + 0J_{2x} + 0J_{2y} + 32J_{11} - 8J_{int} - 16A$
AFM2-AFM	$E_0 + 0J_1 + 0J_{2x} + 0J_{2y} + 32J_{11} + 8J_{int} - 16A$
AFM3-FM	$E_0 + 0J_1 + 0J_{2x} + 0J_{2y} + 32J_{11} - 8J_{int} - 16A$
AFM3-AFM	$E_0 + 0J_1 + 0J_{2x} + 0J_{2y} + 32J_{11} + 8J_{int} - 16A$
AFM4-FM	$E_0 + 0J_1 + 16J_{2x} + 16J_{2y} - 32J_{11} - 8J_{int} - 16A$
AFM4-AFM	$E_0 + 0J_1 + 16J_{2x} + 16J_{2y} - 32J_{11} + 8J_{int} - 16A$
AFM5-FM	$E_0 + 0J_1 + 0J_{2x} + 16J_{2y} + 0J_{11} - 8J_{int} - 16A$
AFM5-AFM	$E_0 + 0J_1 + 0J_{2x} + 16J_{2y} + 0J_{11} + 8J_{int} - 16A$
AFM6-FM	$E_0 + 0J_1 + 16J_{2x} + 0J_{2y} + 0J_{11} - 8J_{int} - 16A$
AFM6-AFM	$E_0 + 0J_1 + 16J_{2x} + 0J_{2y} + 0J_{11} + 8J_{int} - 16A$

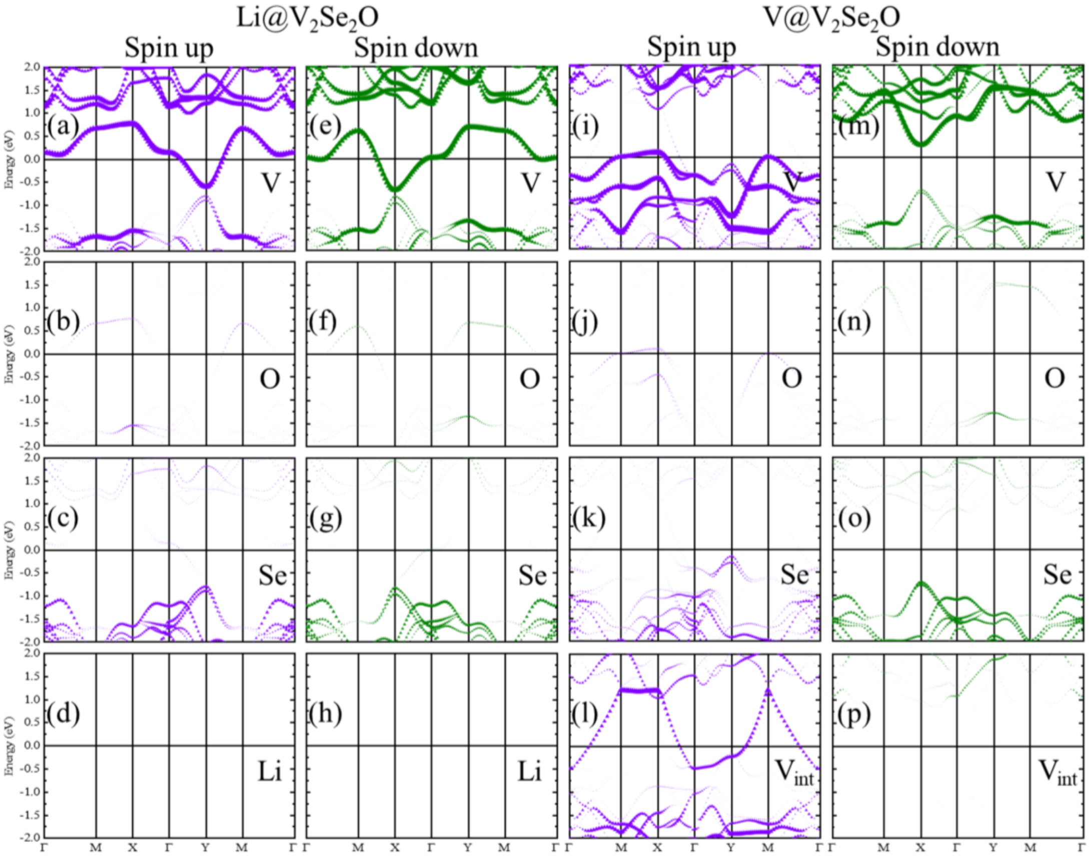

Figure S5. The atom- and spin-resolved band structures for the V_2Se_2O bilayer with Li (a-h) and V (i-p) intercalations, in which V and V_{int} represent the native and intercalated V, respectively.

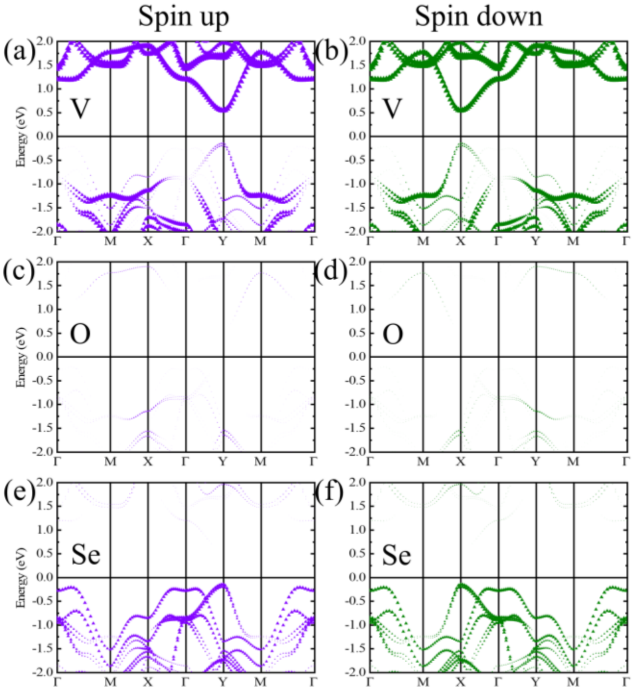

Figure S6. The atom- and spin-resolved band structures for the V_2Se_2O bilayer with no intercalation.

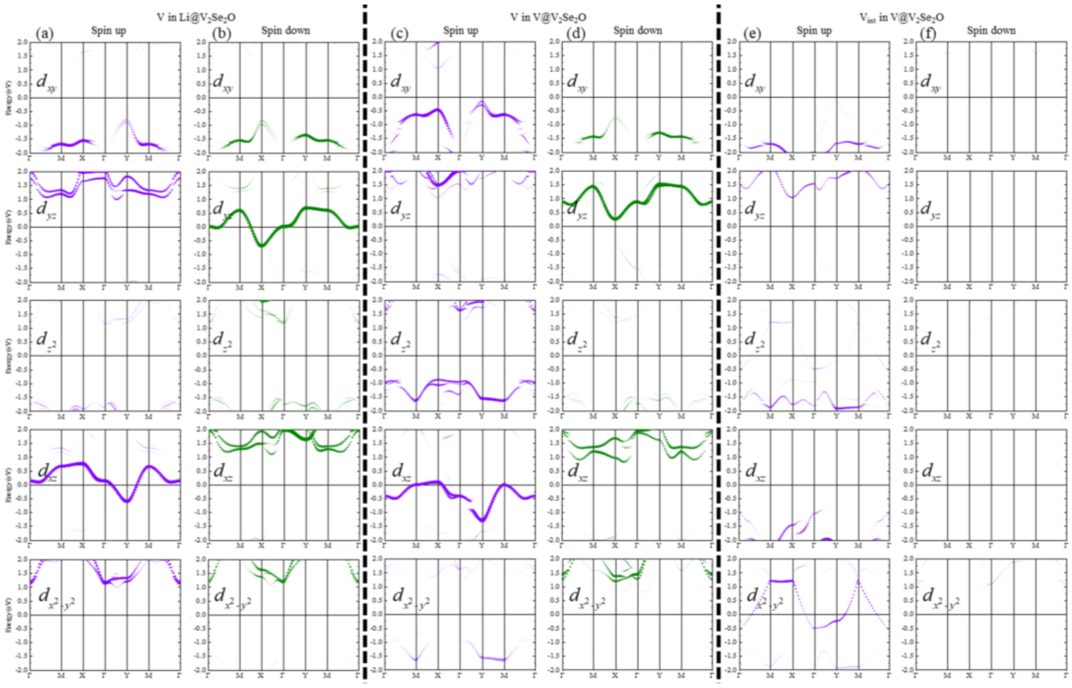

Figure S7. The V-*d*-orbital- and spin-resolved band structures, in which the left, medium and right two columns belong to V atoms in the V_2Se_2O bilayer with Li intercalation, the native, and intercalated V atoms in the V_2Se_2O bilayer with V intercalation, respectively.

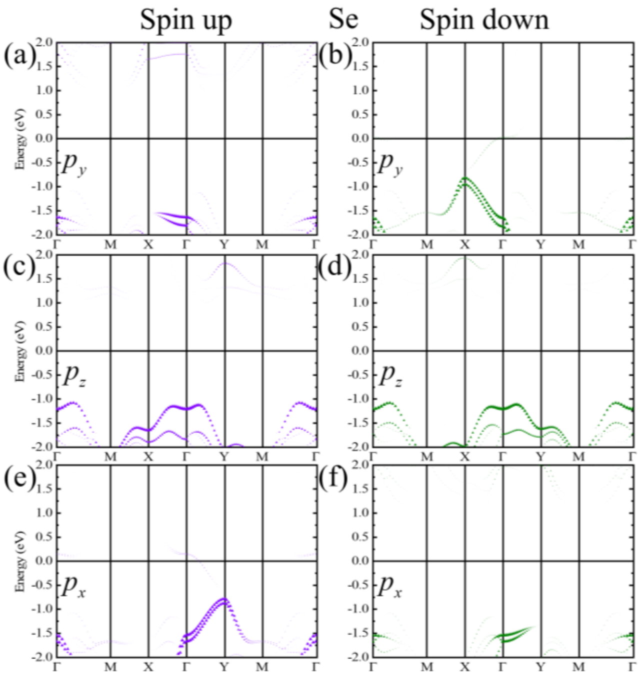

Figure S8. The Se-*p*-orbital- and spin-resolved band structures for the V_2Se_2O bilayer with Li intercalation.

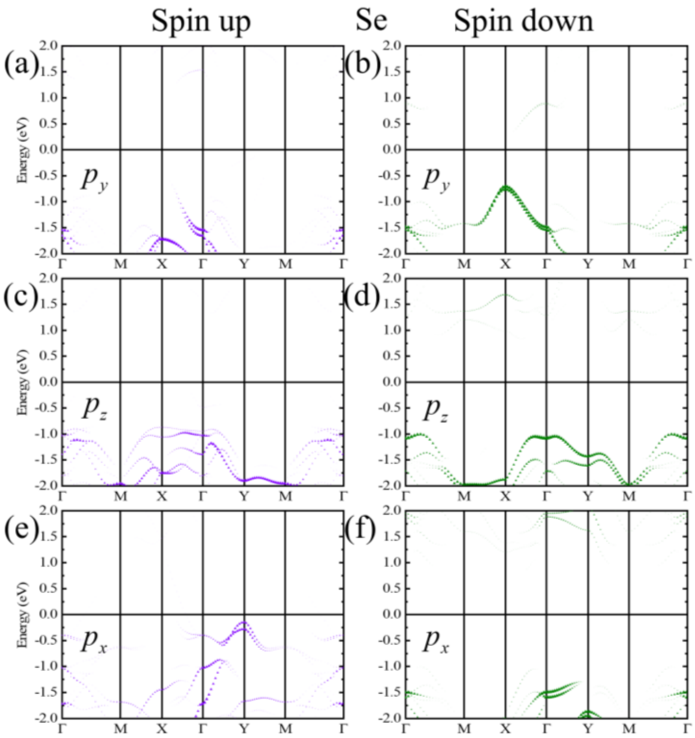

Figure S9. The Se-*p*-orbital- and spin-resolved band structures for the V_2Se_2O bilayer with V intercalation.

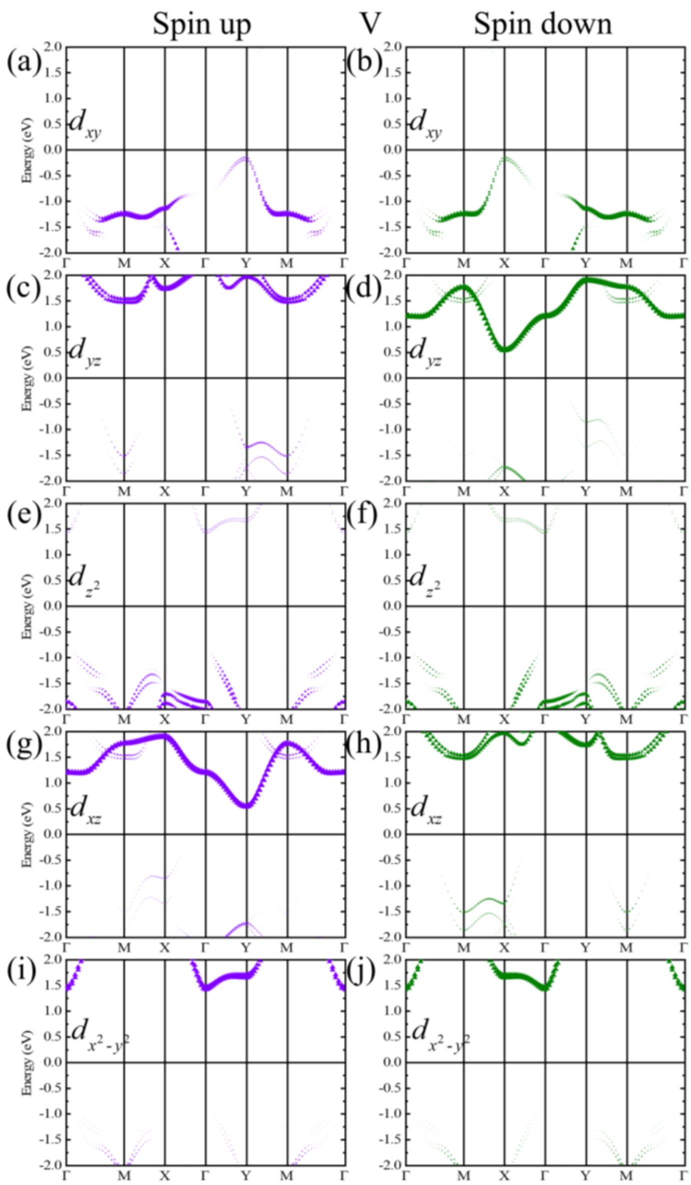

Figure S10. The V-*d*-orbital- and spin-resolved band structures for the V_2Se_2O bilayer with no intercalation.

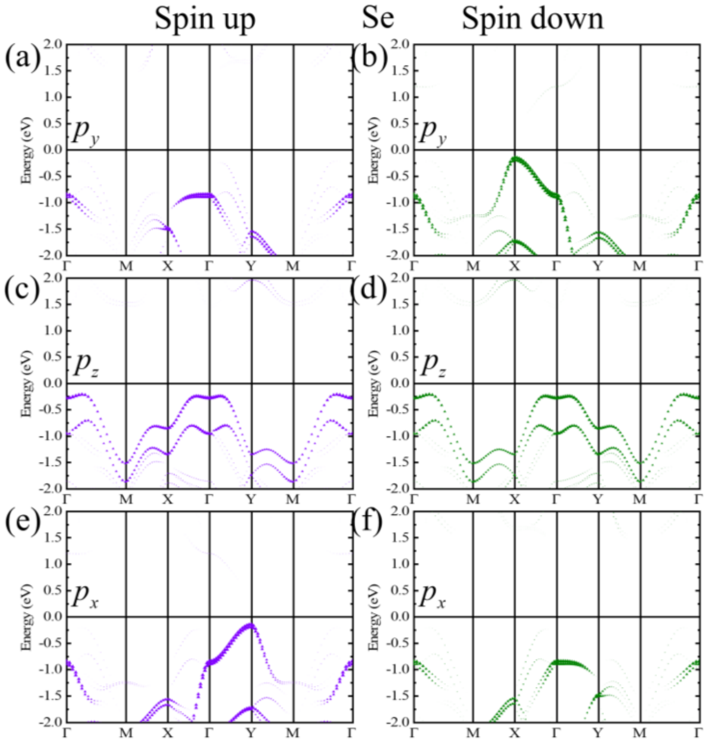

Figure S11. The Se-*p*-orbital- and spin-resolved band structures for the V_2Se_2O bilayer with no intercalation.

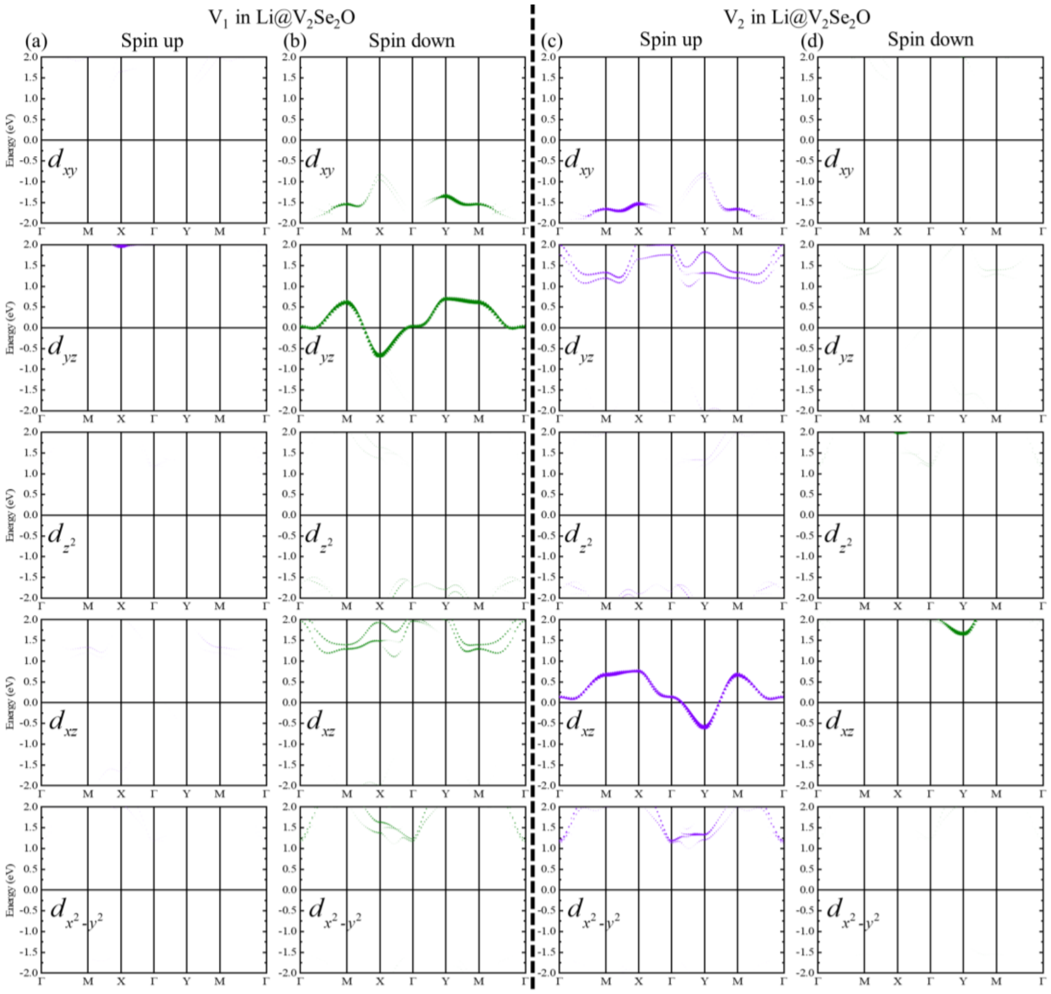

Figure S12. The V_1- (a,b) and V_2- (c,d) *d*-orbital- and spin-resolved band structures for the V_2Se_2O bilayer with Li intercalation.

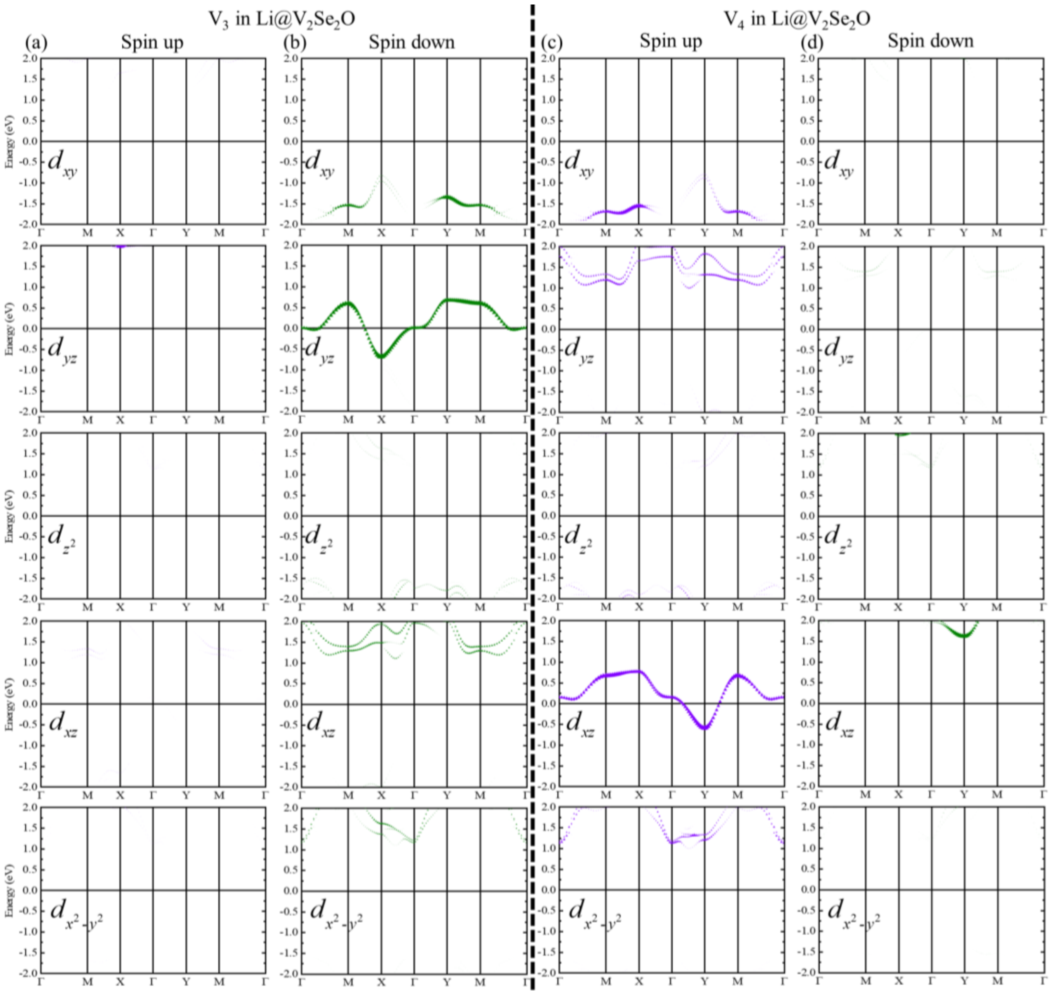

Figure S13. The V_3- (a,b) and V_4- (c,d) *d*-orbital- and spin-resolved band structures for the V_2Se_2O bilayer with Li intercalation.

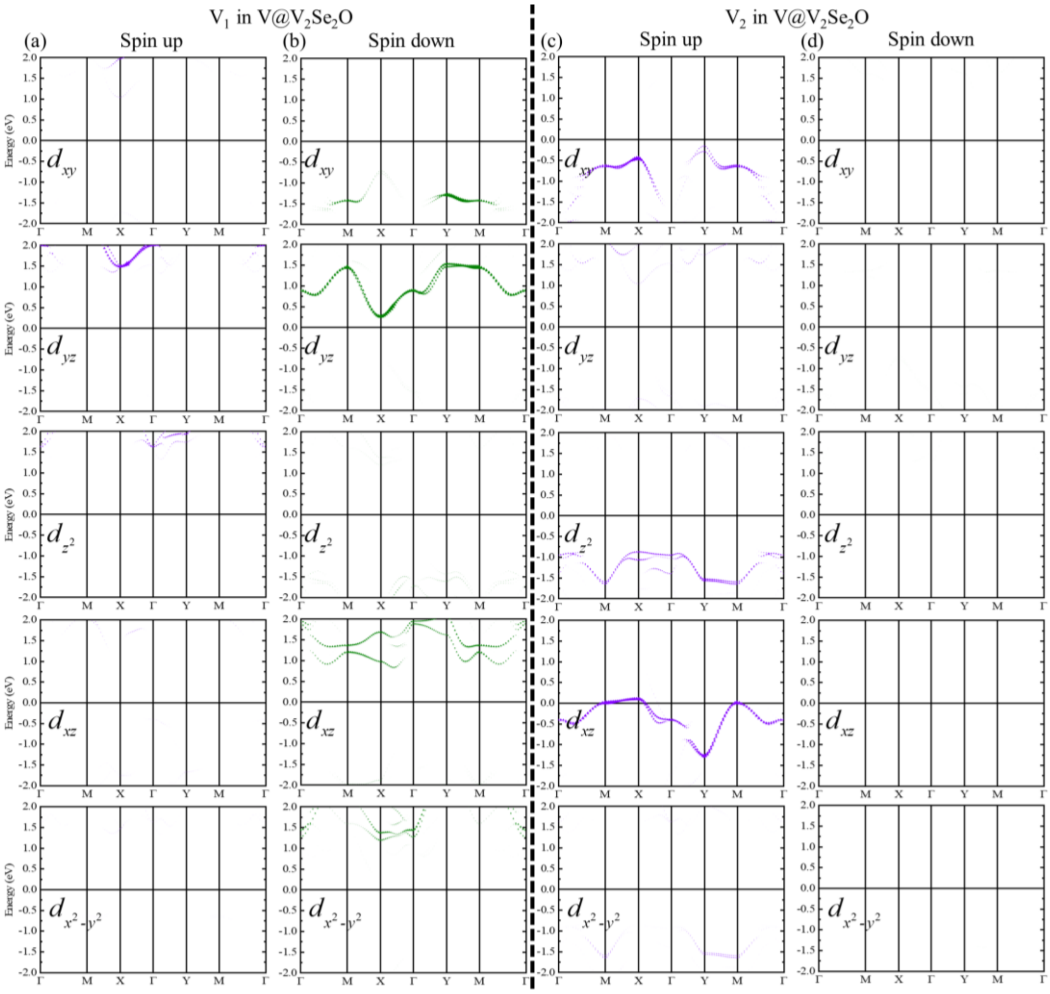

Figure S14. The V₁- (a,b) and V₂- (c,d) *d*-orbital- and spin-resolved band structures for the V_2Se_2O bilayer with V intercalation.

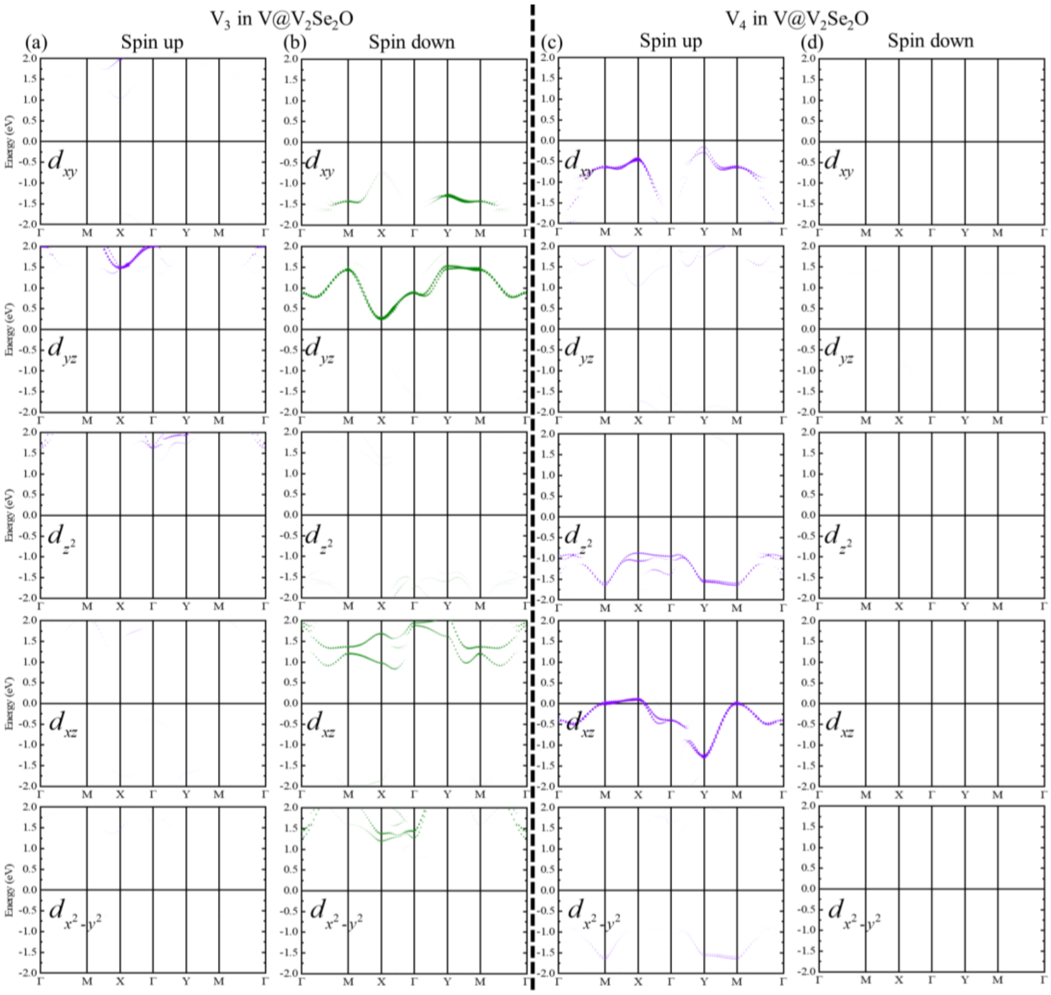

Figure S15. The V_3- (a,b) and V_4- (c,d) *d*-orbital- and spin-resolved band structures for the V_2Se_2O bilayer with V intercalation.

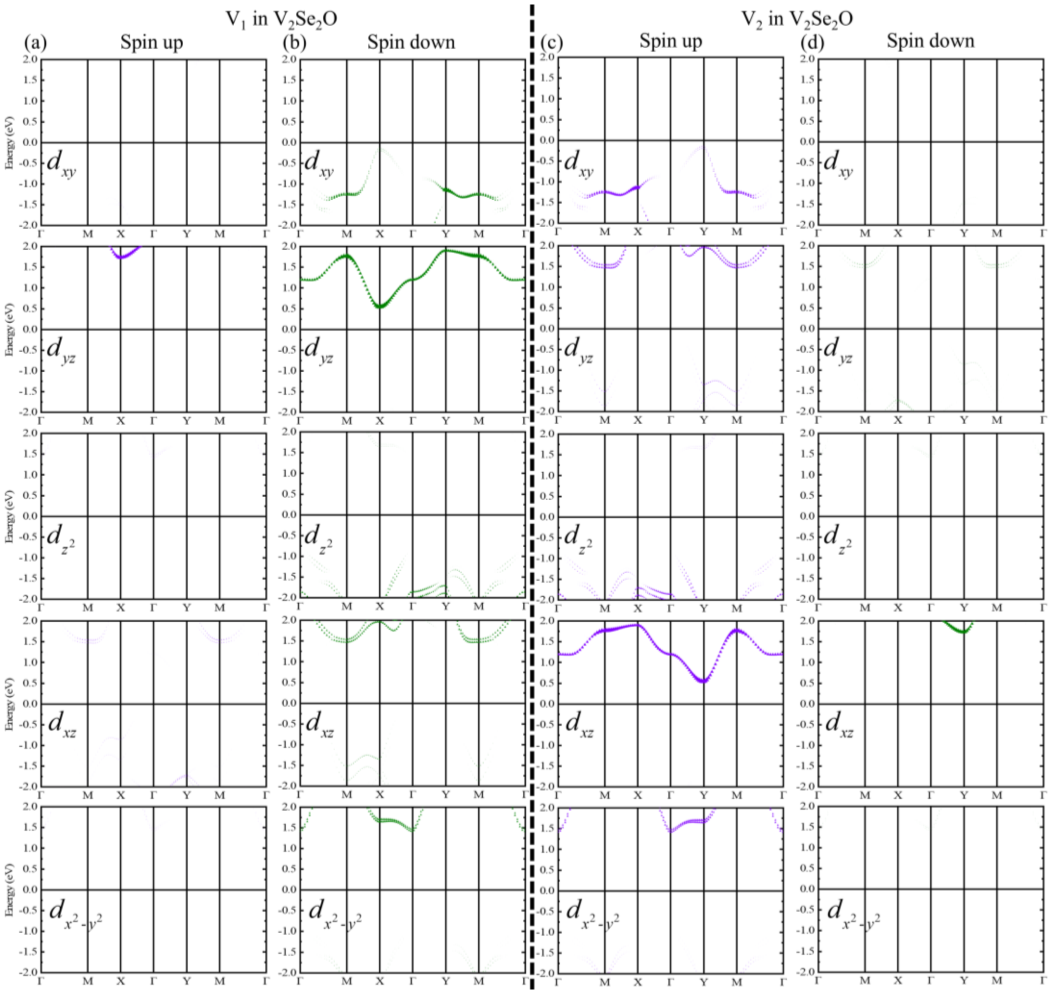

Figure S16. The V$_1$- (a,b) and V$_2$- (c,d) *d*-orbital- and spin-resolved band structures for the V_2Se_2O bilayer with no intercalation.

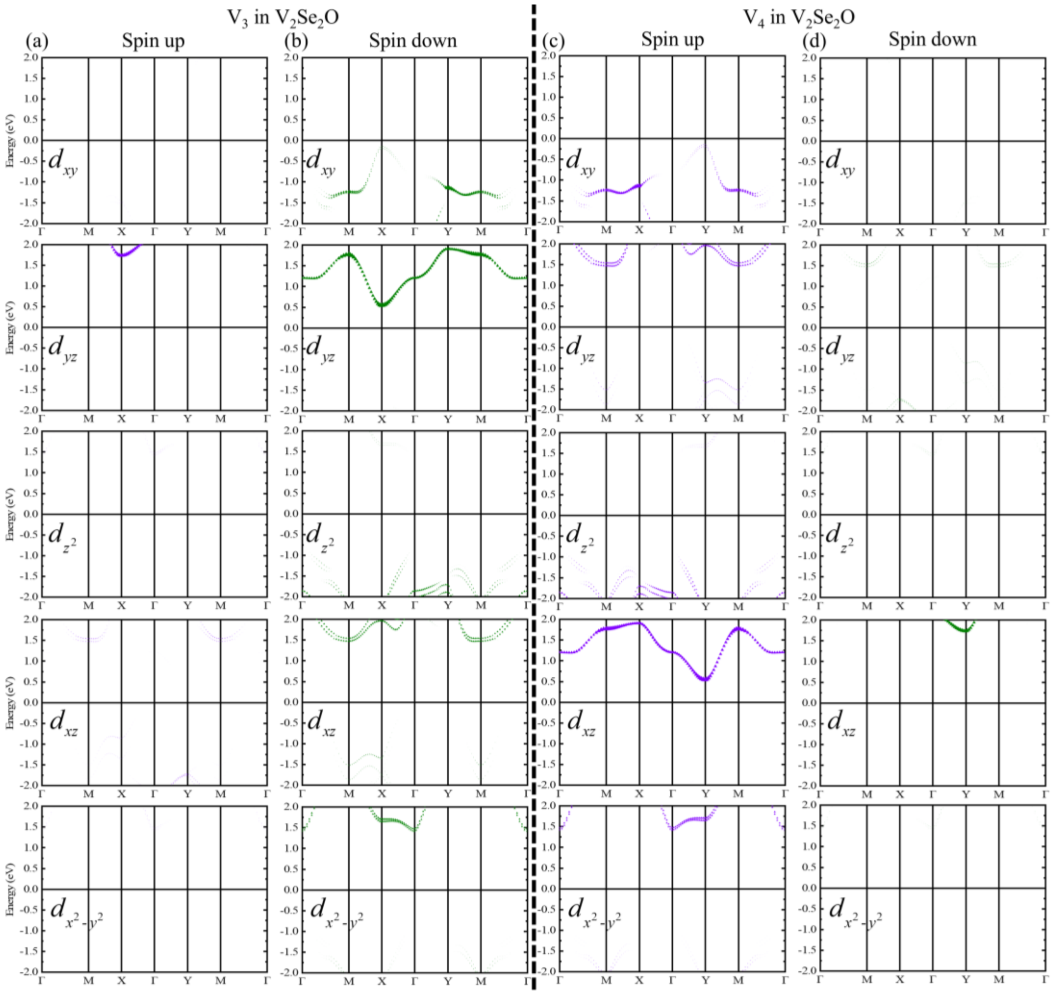

Figure S17. The V_3- (a,b) and V_4- (c,d) *d*-orbital- and spin-resolved band structures for the V_2Se_2O bilayer with no intercalation.

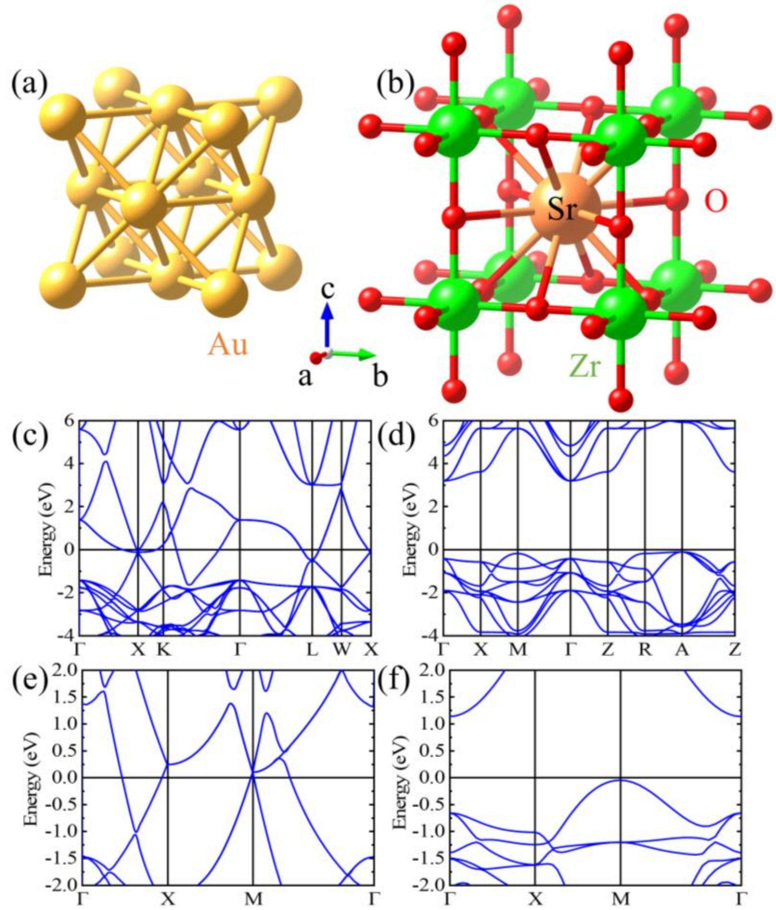

Figure S18. The crystal structures (a,b) and the band structures for bulk (c,d) and monolayer (e,f) of Au and $SrZrO_3$.

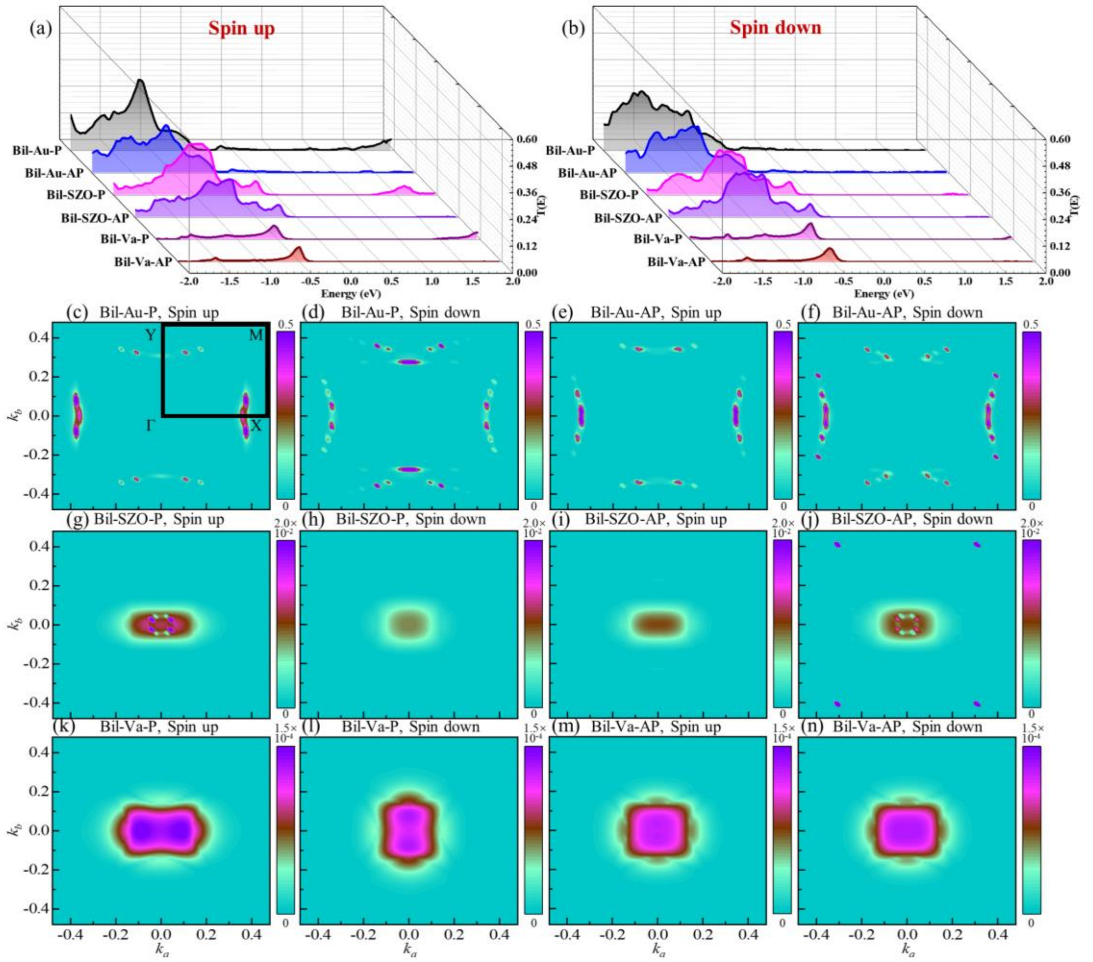

Figure S19. The transmission coefficients as a function of energy in spin up (a) and down (b) channels. The $\vec{k}_{//}$- and spin-resolved transmission spectrum in the 2D Brillouin zone across V_2Se_2O-based MR devices (c-n), in which the types are represented by Bil-Au/SZO/Va-P/AP, Bil stands for V_2Se_2O bilayer with no intercalation, Au/SZO/Va stands for the intermediate layer of Au, $SrZrO_3$ and vacuum, and P and AP stand for the parallel and antiparallel configurations, respectively.

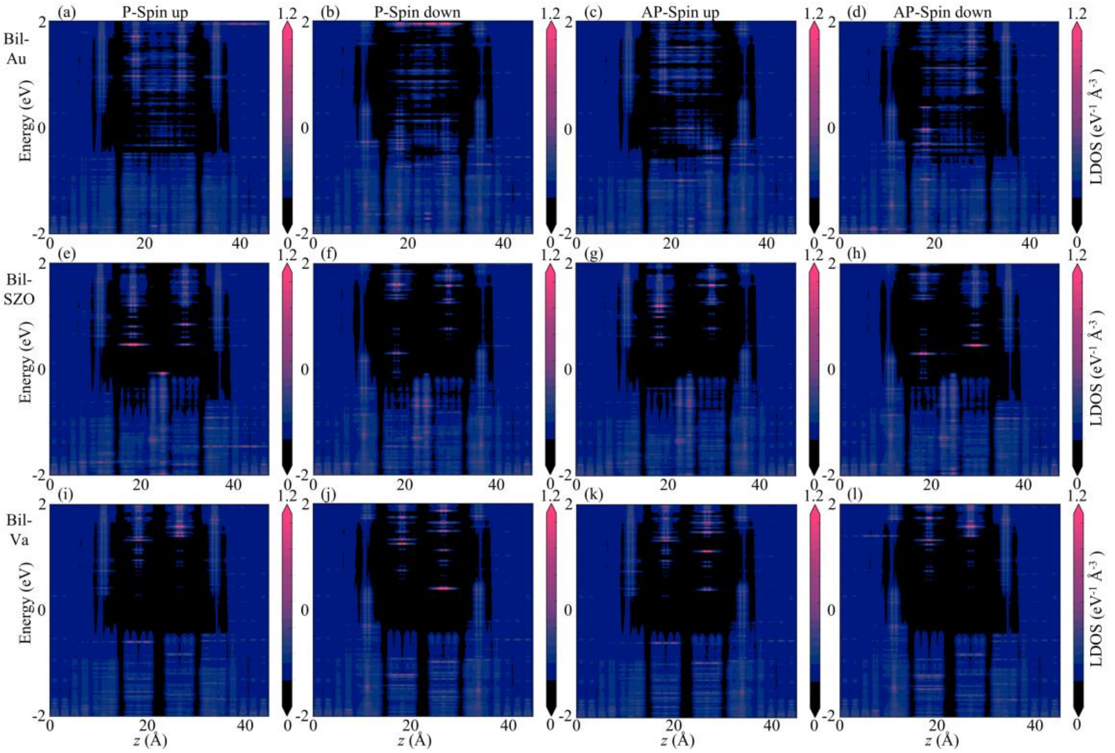

Figure S20. The spin-resolved local density of states (LDOS) across V_2Se_2O-based MR devices (a-x), in which the types are represented by Bil-Au/SZO/Va, Bil stands for the V_2Se_2O bilayer with no intercalation, Au/SZO/Va stands for the intermediate layer of Au, $SrZrO_3$ and vacuum, and P and AP stand for the parallel and antiparallel configurations, respectively.

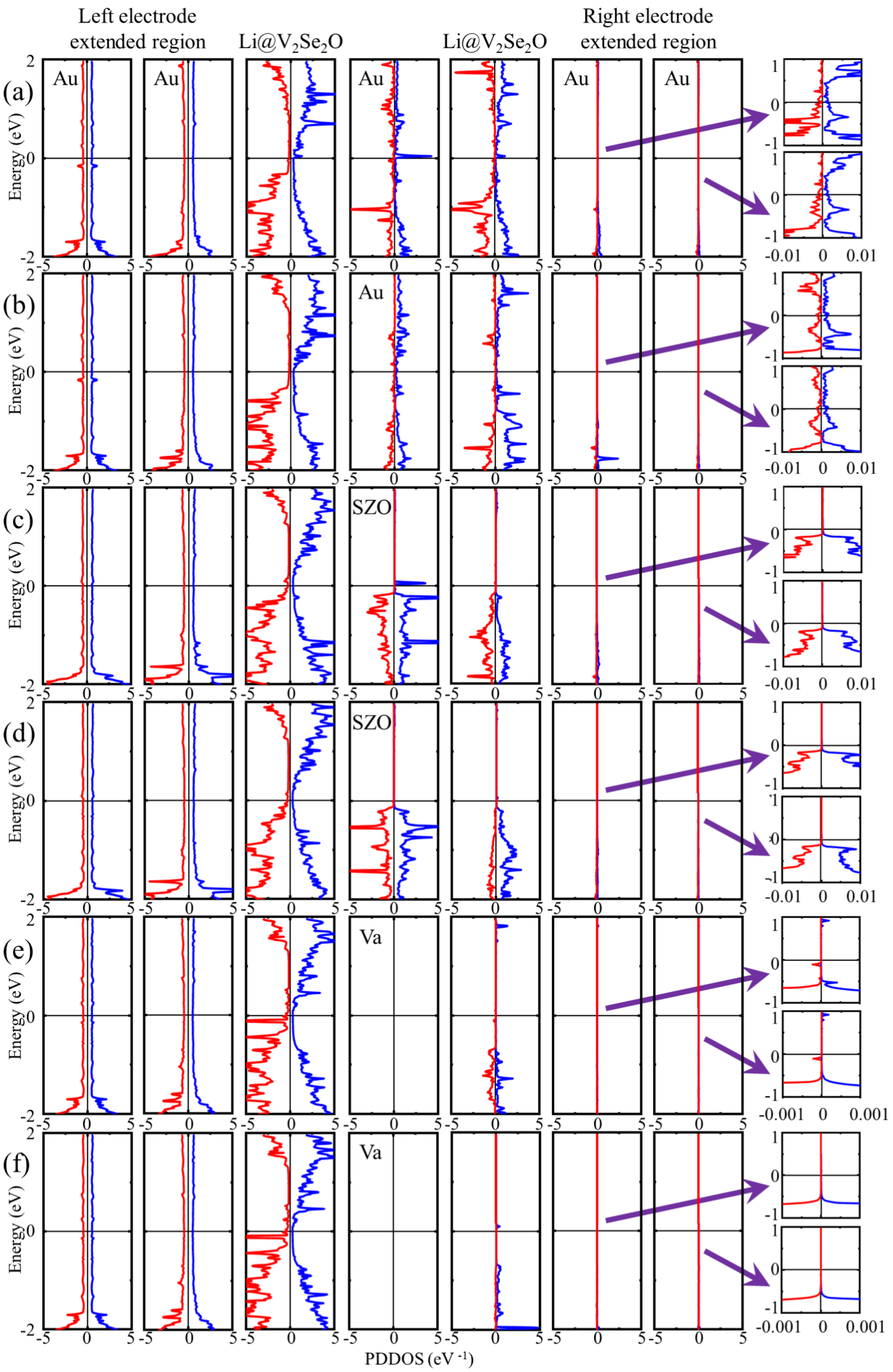

Figure S21. The spin- and layer-resolved projected device density of states (PDDOS) across V_2Se_2O-based MR devices, which is constructed by the parallel and antiparallel V_2Se_2O bilayers with Li intercalation and the intermediate layer of Au (a,b), $SrZrO_3$ (c,d) and vacuum (e,f). The source of contribution is set as the left Au electrodes.

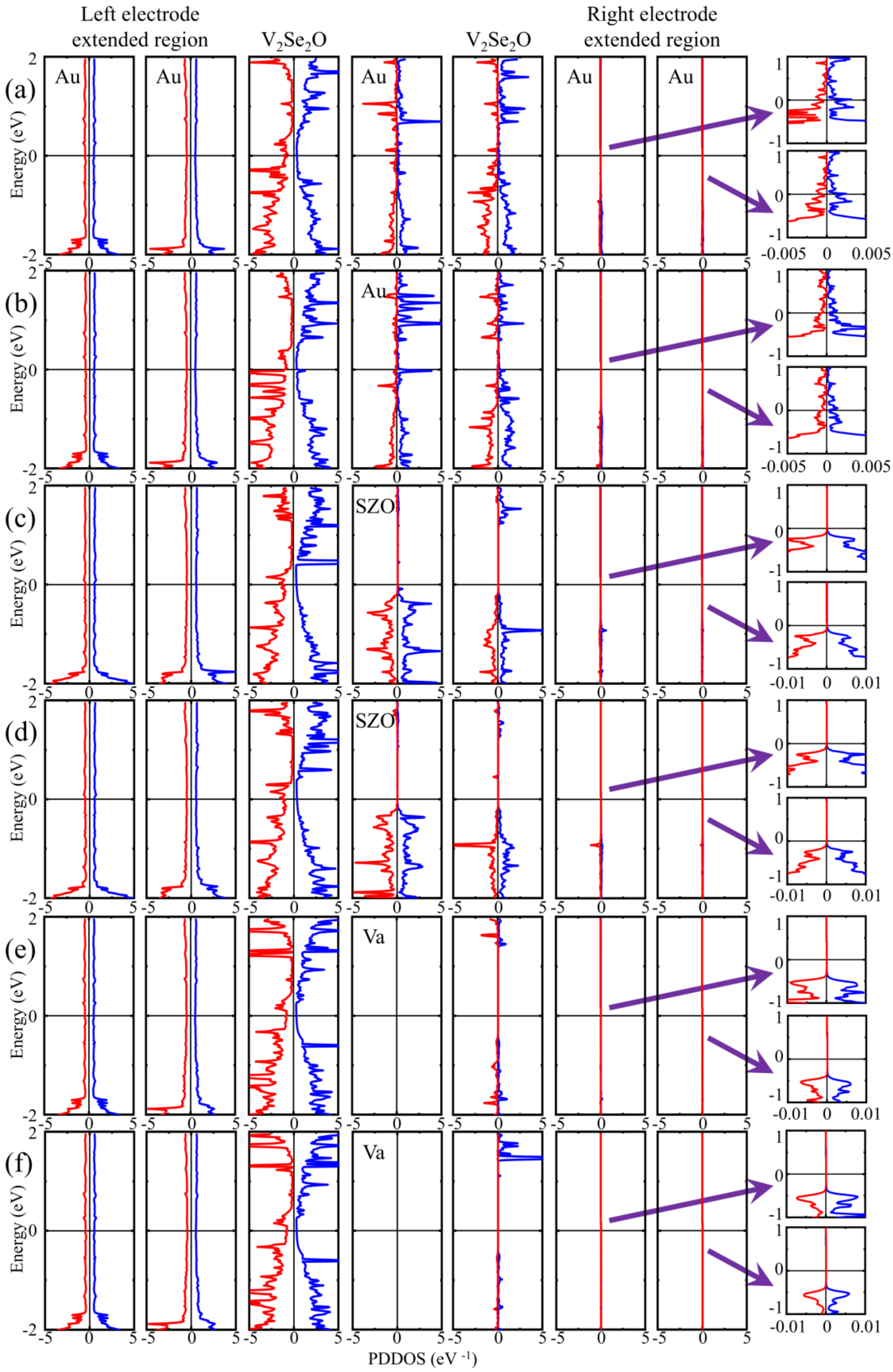

Figure S22. The spin- and layer-resolved projected device density of states (PDDOS) across V_2Se_2O-based MR devices, which is constructed by the parallel and antiparallel V_2Se_2O bilayers with no intercalation and the intermediate layer of Au (a,b), $SrZrO_3$ (c,d) and vacuum (e,f). The source of contribution is set as the left Au electrodes.

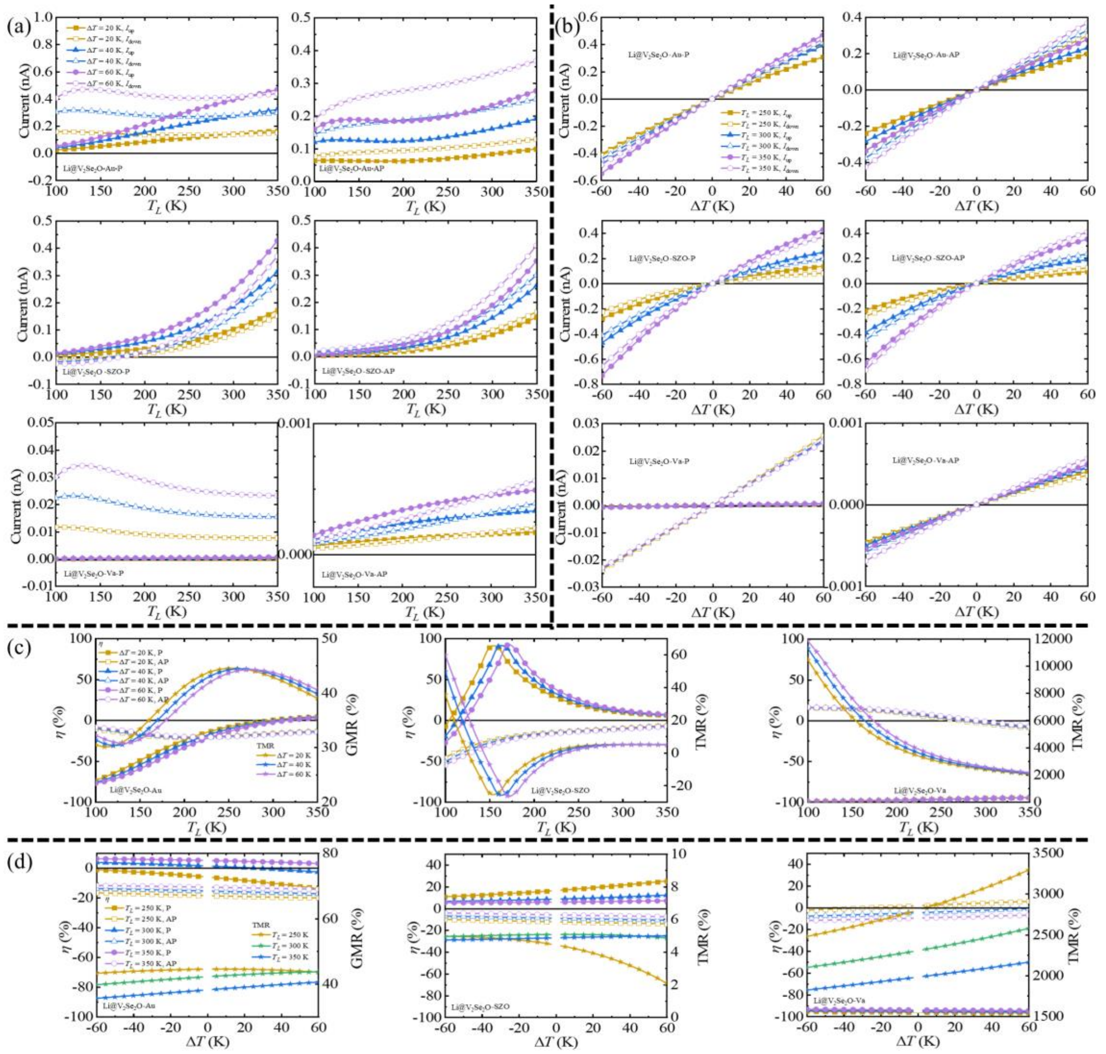

Figure S23. Thermal spin transport performance of V_2Se_2O bilayer with Li intercalation, Au/SZO/Va stands for the intermediate layer of Au, $SrZrO_3$ and vacuum, and P and AP stand for the parallel and antiparallel configurations, respectively. The thermal spin-dependent current, versus T_L and ΔT for different ΔT and T_L (a,b), the thermal spin filtering efficiency η and TMR versus T_L for different ΔT (c) and versus ΔT for different T_L (d).

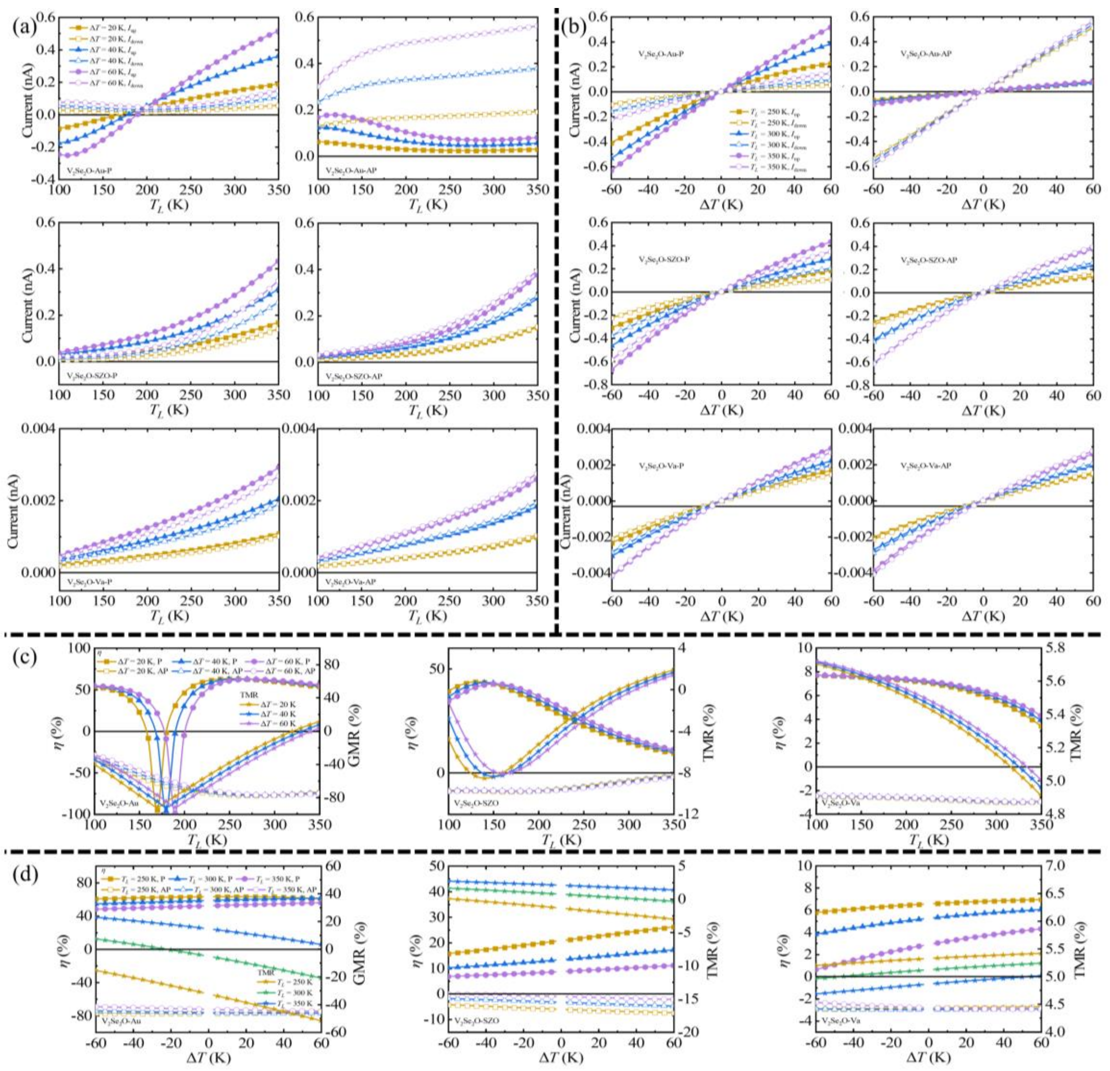

Figure S24. Thermal spin transport performance of V_2Se_2O bilayer with no intercalation, Au/SZO/Va stands for the intermediate layer of Au, $SrZrO_3$ and vacuum, and P and AP stand for the parallel and antiparallel configurations, respectively. The thermal spin-dependent current, versus T_L and ΔT for different ΔT and T_L (a,b), the thermal spin filtering efficiency η and TMR versus T_L for different ΔT (c) and versus ΔT for different T_L (d).
